# Changes in afterload and contractility in patients with severe aortic stenosis after transcatheter aortic valve replacement

**DOI:** 10.1093/ehjimp/qyaf063

**Published:** 2025-05-20

**Authors:** Kristian B Laursen, Rasmus Carter-Storch, Patricia A Pellikka, Mulham Ali, Nils S B Mogensen, Kristian A Øvrehus, Marie-Annick Clavel, Jordi S Dahl

**Affiliations:** Department of Cardiology, Cardiovascular Research Unit, Odense University Hospital, 5000 Odense, Denmark; OPEN, Open Patient Data Explorative Network, Odense University Hospital, 5000 Odense, Denmark; Department of Cardiology, Cardiovascular Research Unit, Odense University Hospital, 5000 Odense, Denmark; Department of Cardiovascular Medicine, Mayo Clinic, Rochester, MN, USA; Department of Cardiology, Cardiovascular Research Unit, Odense University Hospital, 5000 Odense, Denmark; Department of Cardiology, Cardiovascular Research Unit, Odense University Hospital, 5000 Odense, Denmark; Department of Cardiology, Cardiovascular Research Unit, Odense University Hospital, 5000 Odense, Denmark; Department of Cardiology, Cardiovascular Research Unit, Odense University Hospital, 5000 Odense, Denmark; Institut Universitaire de Cardiologie et de Pneumologie, Université Laval, Quebec City, Québec, Canada; Department of Cardiology, Cardiovascular Research Unit, Odense University Hospital, 5000 Odense, Denmark; Department of Cardiovascular Medicine, Mayo Clinic, Rochester, MN, USA

**Keywords:** severe aortic stenosis, echocardiography, computed tomography, hemodynamics, transcatheter aortic valve replacement

## Abstract

**Aims:**

In aortic stenosis (AS), estimation of left ventricular (LV) contractility is difficult as most markers of systolic LV function are load-dependent. The ratio of LV ejection fraction (LVEF) to end-systolic wall stress (ESWS), has been widely accepted as a marker of contractility. However, no studies have evaluated if this ratio is affected by loading conditions. The study describes changes in ESWS and ESWS corrected LVEF after transcatheter aortic valve replacement (TAVR).

**Methods and results:**

In this prospective study, 41 patients with severe AS underwent echocardiography, LV catheterisation, and computed tomography (CT) before and immediately after TAVR. ESWS was estimated from echocardiography alone (ESWS_Echo_), combining CT LV dimensions and echocardiographic gradients (ESWS_CT_  _+_  _echo_) and combining CT LV dimensions and invasively measured LV end-systolic pressure (ESWS_CT_  _+_  _Invasive_). ESWS_echo_, ESWS_CT_  _+_  _echo_ and ESWS_CT_  _+_  _Invasive_ all decreased significantly after TAVR (89 ± 48 vs. 57 ± 37 Kdynes/cm^2^, *P* < 0.01; 69 ± 8 vs. 51 ± 8 Kdynes/cm^2^, *P* < 0.01, and 197 ± 69 vs. 137 ± 48 Kpa/cm^2^, *P* < 0.01, respectively). We observed weak to moderate associations between the methods. After TAVR, LVEF corrected to ESWS_echo_, ESWS_CT_  _+_  _echo_ and ESWS_CT_  _+_  _Invasive_ increased (0.93 ± 0.07 vs. 1.91 ± 2.1, *P* = 0.013; 0.36 ± 0.19 vs. 0.58 ± 0.33, *P* < 0.01, and 0.3 ± 0.02 vs. 2.5 ± 1.5, *P* < 0.01, respectively).

**Conclusion:**

ESWS_echo_, ESWS_CT_  _+_  _echo_ and ESWS_CT_  _+_  _Invasive_ decreased significantly after TAVR suggesting they reflect afterload, but independent of method, ESWS corrected LVEF increased slightly post-TAVR, indicating load dependency.

## Introduction

Increased left ventricular (LV) systolic pressure, one of the hallmarks in aortic stenosis (AS), leads to interstitial fibrosis, LV hypertrophy and changes in LV geometry.^1,2^ Although these changes may help to preserve end-systolic wall stress (ESWS) and LV systolic output in the normal range, they occur at the expense of diastolic alterations that increase filling pressures, ultimately leading to LV systolic dysfunction.^3^ ESWS is considered the gold standard marker of LV afterload, and can be estimated both invasively and non-invasively.^4^

LV ejection fraction (LVEF) is the most routinely used parameter when assessing LV systolic function, and LVEF <50% is considered a class I indication for aortic valve replacement (AVR) in severe AS patients, even in the absence of symptoms.^5,6^ However, LVEF is highly load-dependent^7,8^ and may remain normal despite reduced myocardial contractility,^9,10^ or decline despite preserved contractility when afterload is increased (afterload mismatch).^11^ Markers of LV contractility, a term describing the intrinsic function of the LV myocardium independent of loading conditions, are therefore warranted, as they could add an incremental diagnostic value on top of LVEF to detect subclinical LV dysfunction and may guide the optimal timing of AVR.

ESWS corrected LVEF (LVEF/ESWS) has been suggested to reflect LV contractility,^12,13^ with an inverse linear correlation between ESWS and LVEF in patients with preserved contractility.^14^ However, there is a paucity in data describing whether LVEF/ESWS is truly afterload-independent.

Consequently, the purpose of this study was to examine the immediate changes in LVEF/ESWS in patients with symptomatic severe AS after transcatheter AVR (TAVR), and further to compare ESWS to other markers of LV afterload.

## Methods

### Study design

This was a prospective single centre study and was approved by the local regional scientific ethical committee (S-20190163). All patients provided informed consent before the inclusion in the study. Study data were recorded in RedCap electronic data capture tool hosted at OPEN (Open Patient Data Explorative Network).

### Patients

Patients were prospectively included from June to December 2020 in a consecutive manner, from the heart valve clinic at Odense University Hospital after referral from the regional heart-team conference. Eligible patients where those with symptomatic severe AS [defined as aortic valve area (AVA) ≤ 1 cm^2^ and peak aortic valve velocity (V_max_) > 3.5 m/s and classified as severe by a heart team] referred for TAVR. Only patients undergoing transfemoral TAVR were included. Furthermore, we excluded patients with renal failure (eGFR <45 mL/min/1.73 m^2^), those not able to give a signed informed consent, and those with concomitant moderate or severe valvular disease other than AS at the time of inclusion. All patients underwent a comprehensive diagnostic workup prior to TAVR, including transthoracic echocardiography, clinical evaluation including blood-pressure measurement and cardiac-CT. Echocardiography and cardiac-CT scan were repeated within 24 h after TAVR. Active prescriptions were registered based on patient history and The Danish Nation Prescription Registry.

### Echocardiography

Echocardiograms were performed utilising Philips EPIQ CVx (Koninklijke Philips N.V) or GE Medical Vivid 9 ultrasound systems (GE Medical System, Horten, Norway). All pre- and post-TAVR Images were analyzed offline on IntelliSpace Portal 9.0 (Koninklijke Philips N.V) or EchoPAC PC 08 (GE Medical System, Horten, Norway).

Doppler values were calculated as the average of three cardiac cycles for patients with sinus rhythm and at least five for atrial fibrillation. Stroke volume was calculated by multiplying the LV outflow tract area, measured 5 mm from the aortic annulus and the Doppler velocity time integral measured just proximal to the native annulus and was indexed to body surface area (SVi). A SVi < 35 mL/m^2^ was considered low flow.^15^ AVA was estimated using the continuity equation, peak aortic valve velocity was determined in the acoustic window providing the highest velocity, and mean transvalvular gradient was calculated using the modified Bernoulli equation.^16^ LV dimensions well as biplane Simpson LVEF were measured as recommended by the American Society of Echocardiography.^17^ Presence of low-flow low-gradient and normal-flow gradient AS was defined according to guidelines.^15^ We divided patients into two groups according to LVEF below or above 55%.^18^

### Cardiac-CT

All cardiac-CT were obtained using a Dual Source CT scanner (Siemens Somatom Definition Force, Siemens Healthcare Solutions, Forcheim, Germany) with the following settings: Gantry rotation time 0.28 s, 0.6 mm collimation, acquisition 2× 128 × 0.6 mm, prospectively ECG-triggered with full tube current during systole (200–400 ms) and reduced tube current (20%) during diastole. A temporal resolution of 75 ms made it possible to visualize high quality images for participants with high or irregular heart rate. Images were analysed using SyngoVia webplatform workstation (Siemens Healthcare Solutions, Forcheim, Germany).

The endocardial and epicardial borders were delineated during optimal systole and diastole to estimate the LV end-systolic volume and end-diastolic volume. In the short-axis view, taken at the lowest LV volume, LV wall thickness and LV end-systolic diameter were estimated in a cross-section just below the LV outflow tract. LV wall thickness was defined as the average of end-systolic posterior and interventricular wall thickness. Measurement of LV wall thickness and end-systolic diameter was performed using orthogonal lines.

### Heart catheterisation

All patients underwent left heart catheterisation before and after valve deployment. Transvalvular pressure curves were obtained simultaneously by two diagnostic pigtail catheters connected to a transducer, one in the LV and one in the ascending aorta (AO). Pressure curves were generated utilising Siemens Sensis Vibe (Siemens Healthcare Solutions, Forcheim, Germany).

### Calculation of ESWS

We estimated ESWS using three different methods: ESWS_echo_ was calculated according to previous recommendations using the following equation and measurements from echocardiography^4^:


$$\begin{array}{rl} \mathrm{ESWS} & =0.334\;\times \;(\mathrm{Systolic}\;\mathrm{blood}\;\mathrm{pressure}+\mathrm{Mean}\;\mathrm{gradient})\\ & \times \frac{LV_{\mathrm{end}\text{-}\mathrm{systolic}\;\mathrm{diameter}}}{LV_{\mathrm{wallthickness}}\;\times \;\left(\frac{1+LV_{\mathrm{wall}\;\mathrm{thickness}}}{LV_{\mathrm{end}\text{-}\mathrm{systolic}\;\mathrm{diameter}}}\right)} \end{array}$$

ESWS_CT_  _+_  _echo_ was calculated based on the above formula, fitted to integrate cardiac-CT and echocardiography data, based on a previously published method.^19^ LV end-systolic diameter and LV wall thickness were obtained from cardiac-CT and the aortic valve mean gradient by echocardiography.

ESWS_CT_  _+_  _invasive_ was calculated utilising Young-LaPlace’s law,^20^ and was considered the gold standard, since it included direct measurement of LV pressure:


$$\sigma =\frac{Pr}{2h}$$

where *σ* is endocardial wall stress, *P* is intracavitary pressure, *r* is endocardial radius and *h* is wall thickness. Combining the invasively estimated LV end-systolic pressure (LVESP) and LV dimensions measured by cardiac-CT in the above formula:


$$\mathrm{ESW}S_{\mathrm{CT}+\mathrm{invasive}}=\frac{\mathrm{LVESP}\;\times \;LV_{\mathrm{end}\text{-}\mathrm{systolic}\;\mathrm{diameter}}}{2\;\times \;LV_{\mathrm{wallthickness}}}$$

LV contractility was defined as the ratio of LVEF/ESWS, where LVEF was estimated by echocardiography and ESWS by the three above mentioned methods.^19,21^ End-systolic elastance was calculated by the non-invasive single beat method but utilising invasive LVESP and systolic blood pressure.^22,23^


$$\mathrm{End}\text{-}\mathrm{systolic}\;\mathrm{elastance}\;=\frac{\mathrm{LVESP}\text{-}(E_{\mathrm{Nd}}\times \;\mathrm{Systolic}\;\mathrm{blood}\;\mathrm{pressure}\;)}{\mathrm{SV}\;\times \;E_{\mathrm{Nd}}}$$

Where End is estimated normalized ventricular elastance at arterial end-diastole.

### Statistical analyses

Data are presented as mean and standard deviation, if Gaussian distributed. Normal distribution was tested visually by qq-plots and histograms. Normally distributed data were analyzed using Student’s *t*-test. Non-normally distributed variables were tested by Wilcoxon rank-sum test and presented as median and interquartile ranges. Categorical data were tested with Fishers’ exact test. Pearson’s correlation coefficient was utilized to describe associations between continuous variables. The statistical tests were carried out at a two-sided 0.05 alpha-level of significance. All statistics were applied via STATA/IC 17.0 (StataCorp LP, Texas, USA).

## Results

**Figure 1:**
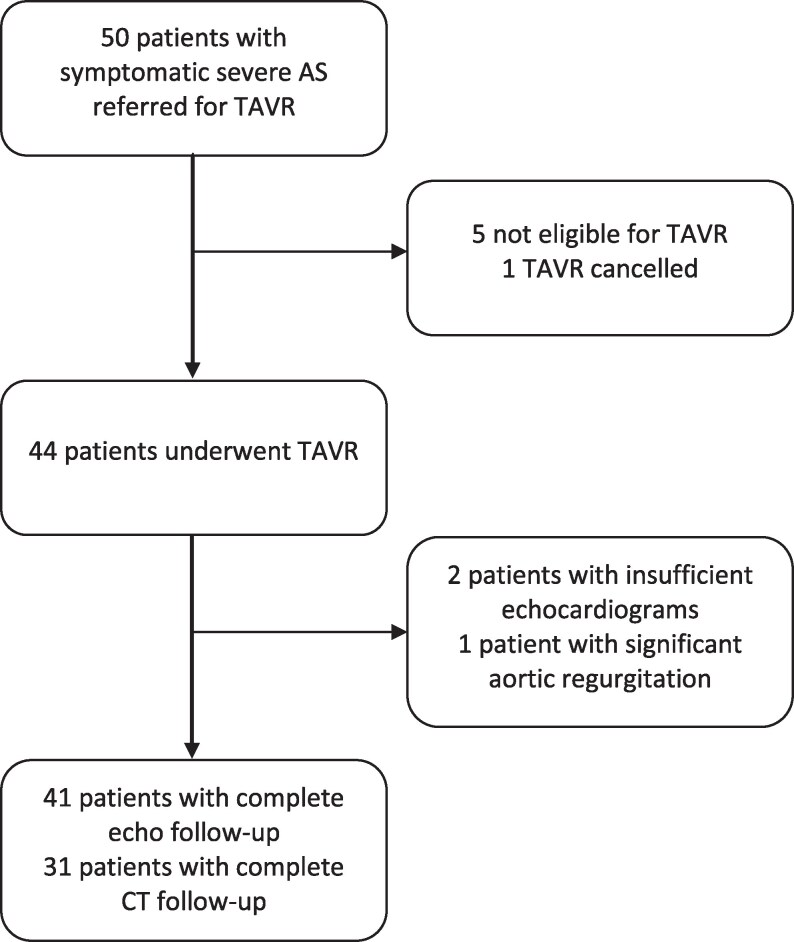
Study inclusion diagram. AS, aortic stenosis; TAVR, transcatheter aortic valve replacement.

Of 50 patients initially screened in the study, nine were excluded (*n* = 5 not suitable for transfemoral TAVR, *n* = 2 poor echocardiographic image quality, *n* = 1 TAVR postponed due to severe pneumonia, and *n* = 1 concomitant moderate aortic regurgitation) leading to the final cohort of 41 patients with complete pre-TAVR work-up. Baseline cardiac-CT was performed at a median of 17 days (IQR 10–21) prior to the baseline transthoracic echocardiography. After TAVR, 10 patients experienced a significant increase in serum creatinine and were not able to undergo post-TAVR contrast cardiac-CT, leaving 31 patients with full evaluation after TAVR (*Figure 1*). Included patients were 81 ± 5 years with male predominance (*n* = 24, 59%). A total of 15 patients (37%) had LVEF <55% at baseline (*Table 1*). There were 31 patients with high-gradient-AS, one with normal-flow low gradient AS, four with paradoxical low-flow low gradient AS and five with classical low-flow low gradient AS.

**Table 1:** Baseline characteristics

	Total	LVEF <55%	LVEF ≥55%	*P*
*n*	41	15	26	
Age (years)	81 ± 5	81 ± 4	81 ± 5	0.88
Sex (male)	24 (59)	10 (67)	14 (54)	0.27
Body surface area (m^2^)	1.9 ± 0.2	1.9 ± 0.3	1.8 ± 0.2	0.84
Syncope	2 (5)	0 (0)	2 (8)	1.00
Angina	15 (37)	5 (33)	10 (38)	0.74
Dyspnea	37 (90)	14 (93)	23 (88)	1.00
Chronic obstructive pulmonary disease	20 (49)	7 (47)	13 (50)	0.84
Type 2 diabetes mellitus	9 (22)	4 (27)	5 (19)	0.70
Hypertension	32 (78)	11 (73)	21 (81)	0.70
Ischaemic heart disease	10 (24)	5 (33)	5 (19)	0.31
Atrial fibrillation	12 (29)	3 (20)	9 (35)	0.32
Heart failure	4 (10)	4 (27)	0 (0)	0.01
Previous stroke or TCI	8 (19)	2 (13)	6 (23)	0.69
eGFR (mL/min/1.73m^2^)	64 ± 14	65 ± 14	64 ± 15	0.90

Data presented as mean ± SD or numbers (percentages).

CCS, Canadian Class Society; NYHA, New York Heart Association; TCI, transient cerebral ischaemia.

### Valvular hemodynamics and cardiac function

Valvular hemodynamics improved after TAVR, with a significant increase in AVA and reduction in aortic mean gradient (*Table 2*). This improvement led to an overall reduction in LV end-diastolic volume (post-TAVR: 79 ± 32 vs. pre-TAVR: 94 ± 38 mL, *P* = 0.004), a decrease in left atrial volume index (33 ± 15 vs. 38 ± 18 mL/m^2^, *P* = 0.04) and a borderline increase in LVEF (57 ± 10 vs. 55 ± 10%, *P* = 0.07). Overall, a significant decrease in LV ejection time (280 ± 36 vs. 314 ± 40 ms, *P* < 0.001) was detected, but SVi (31 ± 8 vs. 33 ± 8 mL/m^2^, *P* = 0.11), and flow rate (210 ± 59 vs. 199 ± 54 mL/s, *P* = 0.31), were unchanged after TAVR.

**Table 2:** Echocardiography and CT—pre- vs. post-TAVR

	Pre-TAVR	Post-TAVR	*P*
*n*	41	41	
**Echocardiography**			
AVA (cm^2^)	0.6 ± 0.2	1.9 ± 0.6	<0.01
Mean gradient (mmHg)	55 ± 17	7 ± 4	<0.01
Systolic blood pressure (mmHg)	140 ± 17	135 ± 23	0.21
LV end-diastolic volume (mL)	94 ± 38	79 ± 32	<0.01
LV end-systolic volume (mL)	44 ± 29	40 ± 25	0.20
LV ejection fraction (%)	55 ± 10	57 ± 10	0.07
End-diastolic interventricular diameter (mm)	12 ± 3	13 ± 3	0.06
LV posterior wall diastolic thickness (mm)	12 ± 2	12 ± 3	0.96
Stroke volume index (mL/m^2^)	33 ± 10	31 ± 8	0.11
LV Ejection time (ms)	314 ± 40	280 ± 36	<0.01
Flowrate (mL/s)	199 ± 54	210 ± 59	0.31
LV mass index (g/m^2^)	109 ± 35	104 ± 33	0.21
LA volume index (mL/m^2^)	38 ± 18	33 ± 15	0.04
Cardiac output (L/min)	4.4 ± 1.3	4.0 ± 1.2	0.04
Heart rate (beats/min)	73 ± 11	71 ± 14	0.14

Computed tomography			
*n*	41	31	
LV end-systolic diameter (mm)	34 ± 7	33 ± 9	0.03
LV end-systolic wall thickness (mm)	17 ± 2	18 ± 2	0.15

Numbers are mean ± SD. Bold *P*-values are statistically significant.

LA, Left atrial; LV, Left ventricular; LVEF, LV ejection fraction.

### Changes in valvular hemodynamics in low and normal LVEF

At baseline, patients with low LVEF < 55% had smaller AVA, lower SVi, and higher LV end-systolic volume (*Table 3*).

**Table 3:** Echocardiography and CT—pre- vs. post-TAVR according to pre-TAVR LVEF

	Pre-TAVR^a^			Post-TAVR		
*n*	15	26		15	26	
**Echocardiography**						
AVA (cm^2^)	0.5 ± 0.2	0.6 ± 0.2	**0**.**04**	2.0 ± 0.7**	1.8 ± 0.6**	0.39
Mean gradient (mmHg)	50 ± 16	58 ± 17	0.19	6 ± 3**	7 ± 4**	0.44
Systolic blood pressure (mmHg)	136 ± 17	142 ± 17	0.29	135 ± 20	135 ± 24	0.90
LV end-diastolic volume (mL)	109 ± 49	85 ± 28	0.10	95 ± 37	70 ± 24**	**0**.**03**
LV end-systolic volume (mL)	61 ± 40	34 ± 14	**0**.**02**	50 ± 32*	35 ± 18	0.10
LVEF (%)	45 ± 10	61 ± 4	**<0**.**0001**	52 ± 13**	60 ± 6	**0**.**04**
LV end-diastolic interventricular diameter (mm)	13 ± 4	12 ± 3	0.76	14 ± 3	13 ± 3*	0.06
LV end-diastolic posterior wall thickness (mm)	12 ± 3	12 ± 3	0.70	13 ± 4	12 ± 2	0.26
LV end-systolic posterior wall thickness (mm)	18 ± 7	18 ± 5	0.86	20 ± 8	18 ± 5	0.41
Stroke volume index (mL/m^2^)	27 ± 9	36 ± 10	**0**.**006**	30 ± 10	31 ± 8**	0.61
LV Ejection time (ms)	306 ± 38	318 ± 40	0.35	274 ± 34*	283 ± 37**	0.45
Flowrate (mL/s)	168 ± 49	213 ± 54	**0**.**01**	215 ± 70	207 ± 53	0.67
LV mass index (g/m^2^)	127 ± 39	99 ± 29	**0**.**02**	116 ± 38*	98 ± 29	0.10
LA volume index (mL/m^2^)	43 ± 18	35 ± 17	0.18	38 ± 17	31 ± 13	0.13
Cardiac output (L/min)	3.8 ± 1.2	4.8 ± 1.3	**0**.**02**	4.1 ± 1.5	3.9 ± 1.0**	0.74
Heart rate (beats/min)	76 ± 11	72 ± 11	0.28	73 ± 12	69 ± 15	0.43

Computed tomography						
*n*	15	26		10	21	
LV end-systolic diameter (mm)	40 ± 10	32 ± 5	**0**.**007**	39 ± 9*	29 ± 4**	**0**.**009**
LV end-systolic wall thickness (mm)	17 ± 3	18 ± 2	0.17	18 ± 3	19 ± 2	0.27

Numbers are mean ± SD. Bold *P*-values are statistically significant.

LA, Left atrial; LV, Left ventricular; LVEF, LV ejection fraction.

^a^Indicates pre-TAVR baseline LVEF.

^*^Indicates *P*-value <0.05 for within-group change from baseline.

^**^Indicates *P*-value <0.005 for within-group change from baseline.

**Figure 2:**
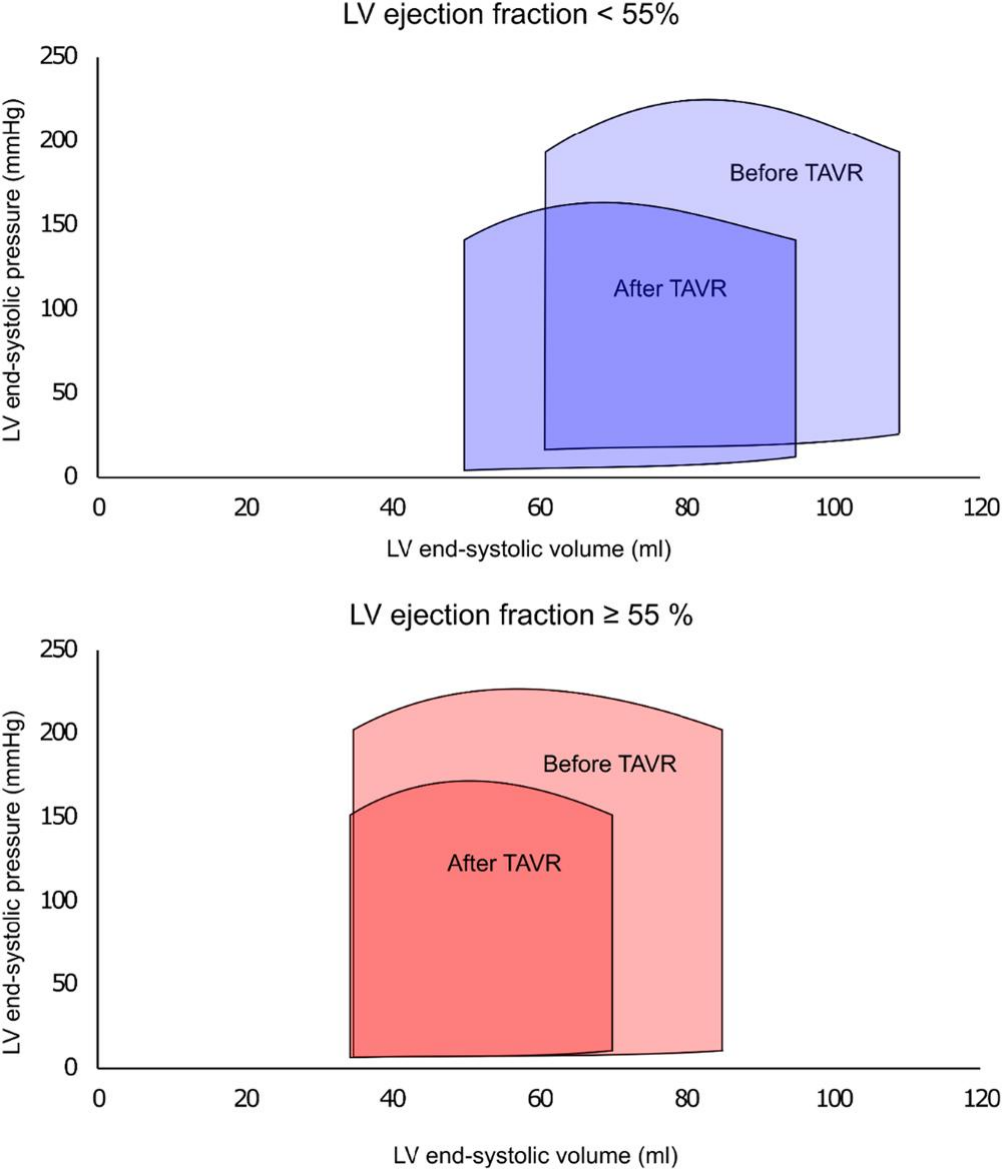
Compiled pressure-volume curves from mean values of invasively measured LV end-diastolic and end-systolic pressures, as well as echo derived LV end-diastolic and end-systolic volumes are shown, illustrating the relationship between afterload reduction after TAVR and changes in LV volume stratified by low LVEF <55% (upper panel) and preserved LVEF ≥ 55% (lower panel) in severe AS patients. Note the reduction in LV stroke volume in the LVEF ≥55% group due to a decrease in LV end-systolic volume with no change in LV end-systolic volume. AS, aortic stenosis; LV, left ventricle; TAVR, transcatheter aortic valve replacement.

Compared with patients with LVEF ≥55%, those with LVEF <55% had a significant decrease in LV end-systolic volume (post-TAVR: 50 ± 32 vs. pre-TAVR: 61 ± 40 mL, *P* = 0.02), leading to an increase in LVEF (52 ± 13 vs. 45 ± 10%, *P* = 0.003). In contrast, patients with LVEF ≥55% had a decrease in SVi after TAVR (31 ± 8 vs. 36 ± 10 mL/m^2^, *P* = 0.002) (*Figure 2*).

### LV pressure and ESWS

ESWS decreased significantly post-TAVR, due to a decrease in LV end-systolic pressure and LV-end-systolic volume, with no change in LV wall thickness (*Tables 2* and *4*). This change was present regardless of the method used (ESWS_Echo_: post-TAVR: 57 ± 37 vs. pre-TAVR: 89 ± 48 Kdynes/cm^2^, *P* < 0.01, ESWS_CT_  _+_  _echo_: 51 ± 8 vs. 69 ± 8 Kdynes/cm^2^, *P* < 0.01, or ESWS_CT_  _+_  _invasive_: 137 ± 48 vs. 197 ± 60 Kpa, *P* < 0.01).

**Table 4:** Changes in LV pressure, ESWS, and ESWS corrected LVEF (LVEF/ESWS) after TAVR procedure

	Pre-TAVR				Post-TAVR			
	Total	LVEF <55%^a^	LVEF ≥55%^a^	*P*	Total	LVEF <55%^a^	LVEF ≥55%^a^	*P*
*n*	41	15	26		31	10	21	
ESWS_echo_ (Kdynes/cm^2^)	89 ± 48	101 ± 54	82 ± 42	0.21	56 ± 36**	65 ± 48*	51 ± 28**	0.30
ESWS_CT_ _+_ _echo_ (Kdynes/cm^2^)	69 ± 8	66 ± 10	70 ± 9	0.17	51 ± 8**	50 ± 7**	52 ± 9**	0.49
ESWS_CT_ _+_ _invasive_ (Kpa)	197 ± 60	235 ± 79	178 ± 38	0.02	137 ± 48**	171 ± 60*	125 ± 36**	0.04
LVEF/ESWS_Echo_	0.6 [0.4–1.3]	0.4 [0.3–1.2]	0.7 [0.5–1.4]	0.03	1.1 [0.8–2.0]**	1.0 [0.4–2.0]*	1.2 [0.9–2.3]**	0.28
LVEF/ESWS_CT_ _+_ _echo_	0.8 [0.7–0.9]	0.7 [0.6–0.8]	0.9 [0.8–0.9]	0.0001	1.1 [1.0–1.3]**	1.1 [0.9–1.3]**	1.2 [1.0–1.3]**	0.35
LVEF/ESWS_CT_ _+_ _invasive_	0.3 [0.2–0.4]	0.2 [0.1–0.3]	0.3 [0.3–0.4]	<0.0001	0.5 [0.4–0.5]**	0.4 [0.3–0.5]**	0.5 [0.4–0.6]**	0.03
End-systolic Elastance (mmHg/mL)	26 [18–32]	32 [28–44]	20 [16–26]	0.0002	18 [15–24]**	21 [17–28]**	17 [15–23]*	0.3550
Invasive peak gradient (mmHg)	72 ± 33	72 ± 30	71 ± 35	0.94	3 ± 3**	3 ± 3**	3 ± 4**	0.65
LV end-diastolic pressure (mmHg)	14 ± 8	21 ± 8	10 ± 4	0.0001	12 ± 6	13 ± 8**	11 ± 5	0.48
LV end-systolic pressure (mmHg)	198 ± 40	193 ± 37	201 ± 42	0.53	147 ± 29**	140 ± 21**	151 ± 32**	0.25
Invasive aortic pressure (mmHg)	127 ± 23	121 ± 21	130 ± 24	0.23	142 ± 29**	137 ± 22**	144 ± 33**	0.48

Numbers are mean ± SD or median [IQR].

ESWS, end-systolic wall stress; LV, left ventricle; LVEF, LV ejection fraction; TAVR, Transcatheter aortic valve replacement.

^a^Indicates pre-TAVR baseline LVEF.

^*^Indicates *P*-value <0.05 for within-group change from baseline.

^**^Indicates *P*-value <0.005 for within-group change from baseline.

Prior to TAVR, there was a weak correlation of ESWS_Echo_ [*r* = 0.36 (95% CI 0.06 to 0.61), *P* = 0.02] and ESWS_CT_  _+_  _echo_ [*r* = 0.07 (95% CI −0.25 to 0.37), *P* = 0.68] with invasively assessed ESWS (ESWS_CT_  _+_  _invasive_). These correlations were similarly weak to non-existent after TAVR: ESWS_Echo_ and ESWS_CT_  _+_  _invasive_ [*r* = 0.17 (95% CI −0.20 to 0.49), *P* = 0.37] and ESWS_CT_  _+_  _echo_ and ESWS_CT_  _+_  _invasive_ [*r* = −0.08 (95% CI −0.42 to 0.28), *P* = 0.68].

### Changes in LV pressure and ESWS according to LVEF group

Before TAVR, ESWS was numerically higher among patients with LVEF <55%, but only statistically different when using the ESWS_CT_  _+_  _invasive_ method (LVEF <55%: 235 ± 79 vs. LVEF ≥55% 178 ± 38 Kpa, *P* = 0.02) (*Table 4*). Accordingly, invasively measured LV end-diastolic pressure was higher in patients with LVEF < 55% (21 ± 8 vs. 10 ± 4 mmHg, *P* = 0.0001).

TAVR resulted in a uniform decrease in LV end-systolic pressure and an increase in aortic pressure regardless of LVEF group. In contrast, only the low LVEF group had a decrease in LV end-diastolic pressure (*Table 4*).

### End-systolic wall stress corrected LVEF (LVEF/ESWS)

**Figure 3:**
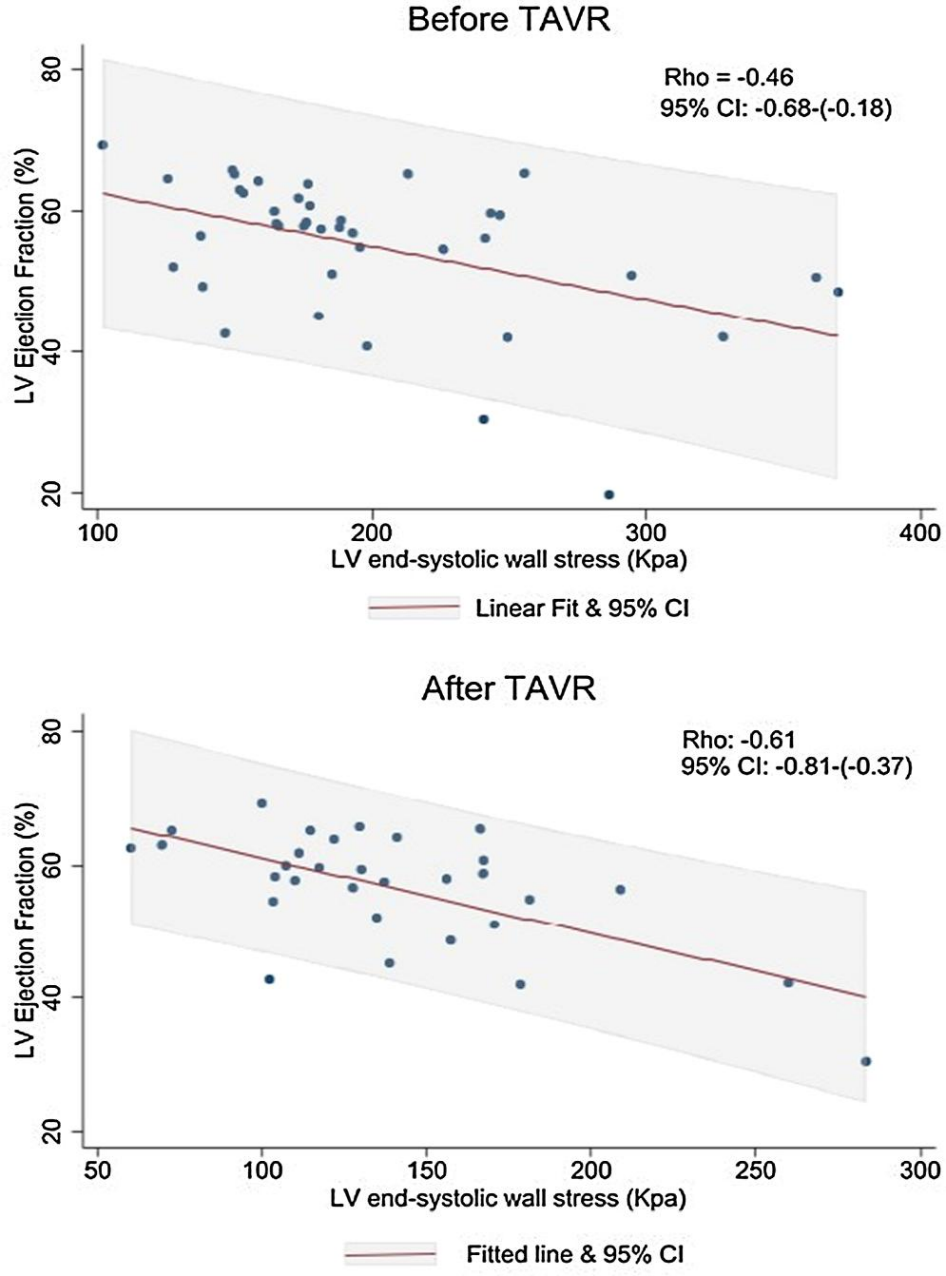
Linear regression models with LV ejection fraction (%) on the *Y*-axis and LV ESWS (KPa) on the *X*-axis, showing a moderate to strong inverse correlation between ESWS_CT_  _+_  _Invasive_ and LVEF, both before and after TAVR. Confirming ealierCorrelation coefficients (rho) and *P*-value are included. ESWS, end-systolic wall stress; LV, left ventricular; TAVR, transcatheter aortic valve replacement.

**Figure 4:**
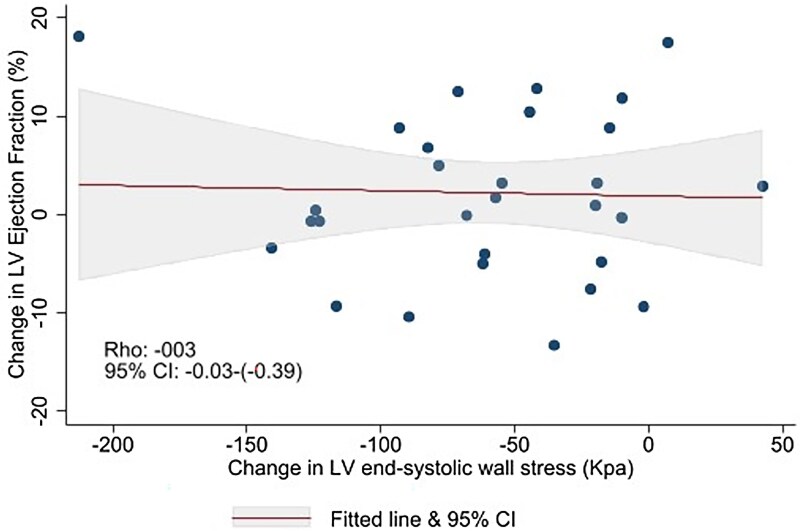
Linear regression model of change in LV ejection fraction (%) on the *Y*-axis and LV ESWS (Kpa) on the *X*-axis before and after TAVR. Showing no significant correlation in of ESWS_CT_  _+_  _Invasive_ and LVEF from before to after TAVR. It suggests no significant association between the degree of afterload reduction and the change in LVEF. The correlations coefficients (rho) and *P*-values are included. ESWS, end-systolic wall stress; LV, left ventricular; TAVR, transcatheter aortic valve replacement.

Although ESWS_CT_  _+_  _Invasive_ showed a moderate to strong inverse correlation with LVEF before TAVR [*r* = −0.46 (95% CI −0.68 to −0.18), *P* = 0.0009] as well as after TAVR [*r* = −0.61 (95% CI −0.79 to −0.33), *P* = 0.0003] (*Figure 3*), the correlation was not significant with ESWS_Echo_ before [*r* = −0.24 (95% CI −0.51 to 0.08), *P* = 0.08] but only after TAVR [*r* = −0.46 (95% CI −0.67 to −0.18), *P* = 0.002] and with ESWS_CT_  _+_  _echo_ only before [*r* = 0.39 (95% CI 0.09 to 0.62), *P* = −0.01] but not after TAVR [*r* = −0.02 (95% CI −0.38 to 0.33), *P* = 0.88]. Further, changes in ESWS_CT_  _+_  _Invasive_ and LVEF from before to after TAVR were not correlated [*r* = −0.03 (95% CI −0.39 to 0.33), *P* = 0.86] (*Figure 4*).

LVEF/ESWS increased significantly regardless of used method: LVEF/ESWS_echo_ (post-TAVR 1.91 ± 2.1 vs. pre-TAVR 0.93 ± 0.68, *P* = 0.01), LVEF/ESWS_CT_  _+_  _echo_ (1.18 ± 0.26 vs. 0.83 ± 0.14, *P* < 0.01), or LVEF/ESWS_CT_  _+_  _invasive_ (2.5 ± 1.5 vs. 0.3 ± 0.02, *P* < 0.01) (*Table 4*).

We performed sensitivity analyses of the changes in LV pressures and ESWS before and after TAVR, excluding patients with atrial fibrillation at the time of analysis, with similar findings (see supplementary data online, *Table S1*).

Additionally, we found end-systolic elastance decreased significantly from pre- to post-TAVR [26 (18–32) mmHg/mL vs. 18 (15–24) mmHg/mL, *P* < 0.005], and according to LVEF groups, LVEF > 55 [20 (16–26) mmHg/mL vs. 17 (15–23), *P* < 0.05] and LVEF ≤ 55 [32 (28–44) mmHg/mL vs. 18 (15–24), *P* < 0.005]. Ees was significantly higher in the lower LVEF group pre-TAVR, while this numerically persisted post-TAVR the difference was not significant (*Table 4*).

## Discussion

In this prospective cohort of patients with severe AS undergoing TAVR, we demonstrate three important findings. (i) LV afterload as measured by ESWS decreased after TAVR. (ii) LVEF did not increase proportionally to ESWS decrease, and therefore LVEF/ESWS increased after TAVR, demonstrating that LVEF/ESWS is afterload-dependent. (iii) Non-invasive ESWS methods showed poor correlation with invasively measured ESWS.

Our findings address the acute haemodynamic changes following TAVR in severe AS, but these changes are preceded by a prolonged period of extensive cardiac remodelling. In early stages of AS, a moderate increase in afterload can usually be compensated by an increase in wall thickness in order to preserve wall stress as well as an increase in preload to maintain stroke volume.^4^ However, at some point, the remodelling process may be insufficient, due to the increasing resistance imposed by the progressively stenotic valve.^24^ Unbalanced, this can result in afterload mismatch and as the valvular disease enters the late stage, the LV may dilate, causing increased ESWS, and consequently reduced LVEF and stroke volume.^25^ Unloading of the LV may improve hemodynamics immediately; in our study we demonstrate an instantaneous decrease in ESWS after TAVR, due to a decrease in both LV end-systolic pressure and LV end-systolic diameter, in line with a previous study.^26^ This decrease in ESWS had several acute haemodynamic effects; mainly diastolic changes, with decreases in LV end-diastolic pressure and volume and left atrial volume index in line with previous studies finding improvement in diastolic function after TAVR.^27,28^ The changes in LV systolic function were more surprising, as we did not observe an overall increase in LVEF after TAVR, but only in the group with LVEF < 55% before TAVR. This is in contrast with previous studies demonstrating an increase in LV global longitudinal strain and LVEF after TAVR.^29,30^ For more than four decades, it has been known that LVEF and ESWS are negatively correlated.^14,24^ We confirm this correlation but extend these findings by demonstrating that ESWS was best measured invasively, whereas our two non-invasive estimations of ESWS showed a weaker negative correlation with LVEF and a weak or non-existent correlation with invasively measured ESWS. However, although the negative LVEF/ESWS correlation was present both before and after TAVR, we did not find that the degree of afterload reduction was associated with a corresponding increase in LVEF.

We demonstrate that SVi declined immediately after TAVR. This counterintuitive phenomenon has previously been described. In a study by Grinberg and colleagues, the authors demonstrated in 97 patients undergoing TAVR that SVi decreased immediately after TAVR but was normalized at 2 month follow-up.^31^ In contrast, a post-hoc study of the PARTNER III trial, showed that SVi only increased slightly (from 41 ± 8 to 42 ± 8 mL/m^2^, *P* < 0.0001) 30 days after TAVR.^32^ Several explanations may partially explain this lack of increase in LVEF and SVi immediately after TAVR. First the decrease in preload we found may have attenuated increase in SVi as AS patients to some extent are preload dependent.^33^ Second, the LV myocardium may be influenced by feedback mechanisms, regulating stroke volume, most notably the ‘shortening deactivation’, a mechanism where a quick shortening of sarcomere length in early systole slows the Ca^2+^ influx into the myocardium in late systole, thus reducing the period of systolic contraction.^34,35^ It is likely that the afterload reduction in TAVR leads to a more effortless early systolic period, which is compensated by a shorter systole. Indeed, we demonstrated that LV ejection time decreased after TAVR, explaining why the flowrate numerically increased after TAVR, despite SVi remaining unchanged. Shortening deactivation could thus explain the lack of increase in LVEF in the patients who already had a normal LVEF before TAVR.

In AS, conventional markers of LV function, such as LVEF, stroke volume and global longitudinal strain may not be indicative of normal myocardial contractility, as they are highly afterload-dependent.^7,36^ Accordingly, LVEF/ESWS has been suggested to be an afterload-independent measurement. It has previously been shown that ESWS is increased while LVEF/ESWS is decreased in symptomatic AS patients,^19^ and LVEF/ESWS could therefore represent an early marker of LV systolic dysfunction. It is therefore surprising that we demonstrate that LVEF/ESWS increased after TAVR as it demonstrates that this marker also is afterload dependent. The change in the LVEF/ESWS relationship was the result of a significant decrease in ESWS without the corresponding increase in LVEF after TAVR this suggests that LVEF/ESWS is dependent on external loading conditions, and therefore does not reflect true intrinsic contractility of the LV.

Thus, it is interesting that we demonstrated a significant reduction in end-systolic elastance, another marker regarded to reflect contractility, as this suggests that even Ees is influenced by haemodynamic changes following TAVR. A key factor contributing to the reduction in Ees, not demonstrated in previous studies,^28,37^ may be the degree of pressure overload as our cohort had a higher aortic valve peak gradient pre-TAVR resulting in higher baseline Ees. Additionally, differences in the distribution of classical vs. paradoxical low-flow, low-gradient AS could further influence the Ees response. However, procedural factors such as rapid ventricular pacing during valve deployment should be considered as this may induce transient myocardial stunning, a factor known to depress LV function acutely.^37,38^

Our findings suggest that contractility assessment remains load-dependent, even with invasive pressure-volume loop analysis and reinforce that ESWS/LVEF indices should not be interpreted as direct markers of myocardial contractility without accounting for loading conditions.

## Clinical implications

While this study highlights the challenges in estimating ESWS non-invasively, invasively measured ESWS is valuable in the clinical evaluation of AS patients. It correlates with LV remodelling, neurohormonal activity, and symptomatic/functional status in AS,^19,26^ and may be an early marker of LV deterioration and poorer outcome. However, although ESWS thus adds insight to the haemodynamic evaluation of AS patients, we demonstrate LVEF/ESWS to be afterload-dependent. Careful consideration of changes in loading conditions should therefore be taken, and non-invasive estimations of ESWS should be evaluated against invasively measured ESWS before being implemented.

## Limitations

Our study was limited to patients with severe AS eligible for transfemoral TAVR, without severe kidney disease. However, we would argue that the hemodynamics of patients with other TAVR approaches and with kidney disease are similar. Due to our inclusion criteria, our patients were mainly patients with high-gradient AS and thus preserved contractility. By choosing these inclusion criteria, we wanted to confirm that our patients had true severe and not moderate-severe AS, but may in doing this have excluded some patients with true severe low-gradient AS. Our findings may therefore not apply to patients with paradoxical low-flow low-gradient with very low gradients.

While pre-TAVR echocardiograms were performed on the day of the procedure, pre-TAVR cardiac-CTs were not obtained at the same time. Post-TAVR echocardiography was conducted on the same day, immediately after the patient left the cath lab, and cardiac CT was repeated within 24 h of TAVR. It is thus possible that this time difference has introduced uncertainty to our evaluation of ESWS_CT_  _+_  _Echo_ and may explain that it demonstrated the weakest association with LVEF.

Additionally, patients with severe AS have multiple factors influencing their myocardial condition. The TAVR procedure itself may have impacted the myocardium function through temporary stunning caused by transient ischaemic damage and rapid pacing during valve deployment.

## Conclusion

Although ESWS decreased after TAVR, LVEF did not increase accordingly, and thus LVEF/ESWS is not load independent as previously believed and cannot be used as a marker of true LV contractility. Non-invasive markers of ESWS show poor agreement with invasively measured ESWS.

## Supplementary Material

qyaf063_Supplementary_Data

## Data Availability

All study-related documents will be made available on request. Individual data collected for the study will be made available for collaborative pooled analyses provided relevant contracts and data sharing agreements are made. Only anonymized data will be shared. Any requests for data access should be directed to the sponsor at Odense University via email: jordi.dahl@rsyd.dk.
