# Supplementary material for: Changes in afterload and contractility in patients with severe aortic stenosis after transcatheter aortic valve replacement
Source: Eur Heart J Imaging Methods Pract. 2025 May 20;3(1):qyaf063. doi: 10.1093/ehjimp/qyaf063 (PMC12142310; doi:10.1093/ehjimp/qyaf063)
Supplement: qyaf063_Supplementary_Data [file qyaf063_supplementary_data.docx]

# Supplementary

| **Supplementary table 1**: Changes in LV pressure, ESWS, and ESWS corrected LVEF after TAVR procedure, only patients with SR at the time of echocardiography | | | | | | | | |
| --- | --- | --- | --- | --- | --- | --- | --- | --- |
|  | **Pre-TAVR** | | | | **Post-TAVR** | | | |
|  | **Total** | **LVEF <55%**^ⴕ^ | **LVEF ≥55%**^ⴕ^ | **p** | **Total** | **LVEF <55%** ^ⴕ^ | **LVEF ≥55%** ^ⴕ^ | **p** |
| N | **34** | **13** | **21** |  | **26** | **8** | **18** |  |
| ESWS_echo_ (Kdynes/cm^2^) | 82±45 | 112±14 | 82±10 | 0.084 | 51±28** | 72±54 | 53±30** | 0.27 |
| ESWS_CT+echo_ (Kdynes/cm^2^) | 71±9 | 66±10 | 71±10 | 0.15 | 52±9** | 50±8** | 52±9** | 0.44 |
| ESWS_CT+invasive_ (Kpa) | 181±42 | 246±79 | 180±41 | 0.0035 | 119±31** | 184±60** | 126±38** | 0.007 |
| LVEF/ESWS_Echo_ | 0.6± [0.4-1.3] | 0.4 [0.3-0.7] | 0.8[0.5-1.4] | 0.10 | 1.1 [0.8-2.0]* | 0.7 [0.4-4.0] | 1.1 [0.9-2.2]* | 0.38 |
| LVEF/ESWS_CT+echo_ | 0.8 [0.7-0.9] | 0.7 [0.6-0.8] | 0.9 [0.8-0.9] | 0.0002 | 1.2 [0.9-1.3]** | 1.1 [0.8-1.4] | 1.2 [1.0-1.4]** | 0.58 |
| LVEF/ESWS_CT+invasive_ | 0.3 [0.2-0.4] | 0.2 [0.1-0.3] | 0.3 [0.3-0.4] | 0.0002 | 0.4 [0.3-0.6] | 0.3 [0.2-0.5]* | 0.5[0.4-0.6]** | 0.01 |
| Invasive peak gradient (mmHg) | 75±38 | 75±31 | 74±38 | 0.98 | 4±4** | 2±3** | 4±4** | 0.23 |
| LV end-diastolic pressure (mmHg) | 10±4 | 28±8 | 9±4 | <0.0000 | 12±5 | 12±8** | 11±5 | 0.58 |
| LV end-systolic pressure (mmHg) | 204±45 | 195±39 | 204±45 | 0.53 | 150±33** | 147±20** | 156±138** | 0.52 |
| Invasive aortic pressure (mmHg) | 130±25 | 120±20 | 130±25 | 0.25 | 146±34* | 145±21** | 147±36** | 0.53 |
| Numbers are mean±SD or median [IQR]. | | | | | | | | |
| Abbreviations: ESWS = end-systolic wall stress, LV = left ventricle, LVEF = LV ejection fraction, TAVR = Transcatheter aortic valve replacement | | | | | | | | |
| ⴕ indicates Pre-TAVR baseline LVEF  ** Indicates p-value <0.005 for within-group change from baseline, * indicates p-value <0.05 for within-group change from baseline | | | | | | | | |
